# FEM-Based Thermogram Correction for Inconel 625 Joint Hardness Clustering

**DOI:** 10.3390/ma15031113

**Published:** 2022-01-31

**Authors:** Wojciech Jamrozik, Jacek Górka, Bernard Wyględacz, Marta Kiel-Jamrozik

**Affiliations:** 1 Department of Fundamentals of Machinery Design, Silesian University of Technology, Konarskiego Str. 18a, 44-100 Gliwice, Poland; 2 Department of Welding Engineering, Silesian University of Technology, Konarskiego Str. 18a, 44-100 Gliwice, Poland; jacek.gorka@polsl.pl (J.G.); bernard.wygledacz@polsl.pl (B.W.); 3 Department of Biomaterials and Medical Devices Engineering, Silesian University of Technology, Roosevelta Str. 40, 41-800 Zabrze, Poland; marta.kiel-jamrozik@polsl.pl

**Keywords:** welding, TIG, thermography, reflected temperature, hardness, clustering

## Abstract

Assessing the temperature of the joint in on-line mode is a vital task that is demanded to characterize the formations of terns formations that are taking place in a joint and result in reaching necessary properties of the joint. Arc welding generates a high amount of heat that is reflected by the metallic surface of the welded object. In the paper, a temperature measurement credibility increase method is described and evaluated. The proposed method is used to reduce the influence of the reflected temperature of the hot torch and the arc on the temperature distribution observed on the surface of the welded joint using an infrared camera. The elaborated approach is based on comparison between infrared observation of the solidifying weld and precisely performed finite element method (FEM) simulation. The FEM simulations were calibrated according to the geometry of the fusion zone. It allows to precisely model heat source properties. The best-reflected temperature correction map was selected and applied to obtain a temperature representation that differs from the FEM baseline by less than 10 °C. Precise temperature values allowed us to cluster welded joints in 3D feature space (temperature, hardness, linear energy). It was found that by using the k-means clustering method it is possible to distinguish between correct and faulty (in terms of too low mechanical properties) joints.

## 1. Introduction

Inconel nickel-chromium superalloys contain approximately 15 to 20% chromium and iron additives up to approximately 18%, molybdenum up to approximately 16%, niobium up to approximately 5% and other elements (Co, Cu, W). They are characterized by high corrosion resistance and high durability at temperatures up to approximately 1000 °C. They are used in the most thermally loaded parts of jet engines, accounting for nearly 50% of their mass [1]. Inconel 625 (EN 2.4856-NiCr22Mo9Nb) being a trademark of the Special Metals Corporation group [2] is a nickel-chromium-molybdenum alloy with an addition of niobium that acts with the molybdenum to stiffen the alloy matrix and thereby provide high strength without strengthening heat treatment. This alloy resists a wide range of severely corrosive environments and is especially resistant to pitting and crevice corrosion. Used in chemical processing, aerospace and marine engineering, pollution control equipment, and nuclear reactors. Inconel 625 is a solid solution-reinforced superalloy. Additionally, strengthening of this material may be derived by precipitation of carbides or intermetallic phases [3]. The solidus temperature of Inconel 625 is 1290 °C and the liquidus temperature is 1350 °C [4].

In the welding of nickel-based alloys, alloy elements segregate mainly at grain boundaries and form low melting point phases such as eutectic γ–γ′, Laves phase, and MC carbides along solidified grain boundaries. The presence of intergranular liquid films results in microfissures because the liquid films cannot withstand the thermal and mechanical tensile stresses generated in the gas tungsten arc welding (GTAW) process. Consequently, solidification cracking and liquation cracking occur due to the solid–liquid interface separation in the brittle temperature range (BTR), which is lower than the melting point of nickel-based alloys. It is one for the critical factors of high hot cracks. Furthermore, there is a ductility dip temperature range (DTR) between solidus temperature (T_S_) and 0.5T_S_ [5]. To perform the welding process correctly, it is necessary to know exact values of temperature.

As Inconel 625 material is a rather expensive one and is often applied in critical applications, it is necessary to ensure the high quality of any made joints. It is especially desirable to use on-line/in-line monitoring methods to reduce/avoid post production quality checks that can result in revealing of systematic weld failures in large production batches. To allow this type of monitoring, a credible, properly calibrated measurement method is needed. One of such approaches can consist of temperature measurements using infrared (IR) cameras. The application of thermography (IR imaging) to monitor and diagnosing welding and welded joints is an engineering and scientific task that has been explored for more than sixty years. The concept of infrared application for nickel alloy welding monitoring emerged in 1966 when work on establishing relationships between infrared radiation (IR) emitted during the weld pulse cycle and the relative tensile strength of the weld was started [6]. Practical considerations on passive and active thermography applications for the evaluation of weld quality were described in the 1970s when commercial devices for IR observation became available [7]. Several interesting phenomenon were found covering connection between temperature and process parameters. One of findings was that current plays an important role in cooling rates (measured with IR camera) and tensile strength of welded joint [8]. Also IR detection was applied to control the welding process by measuring the width of the weld bead, the depth of penetration and the position of the torch [9,10]. However, the main branch of the developed methods is devoted to online monitoring of the weld geometry [11,12] and the temperature distribution in the welding pool [13] using a passive approach. Various image processing techniques and novelty/anomaly detection algorithms were utilized to detect weld defects. Even in many promising results achieved, thermography remains a method that is rarely applied in an industrial environment.

The use of an infrared camera for welding process monitoring is relatively simple, but it is a non-trivial task to get measurement results that are valid. This is due to the nature of infrared radiation. The IR-based temperature measurement is an indirect one, and several factors must be set to calculate temperature from the infrared radiation emitted by an object. Additionally, the welding process requires a large amount of heat that is needed to melt the edges of material pieces to produce the joint. One of the welding techniques, that is often applied for the joint of a wide group of metallic materials is tungsten inert gas (TIG). It can be applied to join dissimilar materials. Nickel alloys can also be welded with other iron alloys, e.g., nonalloy steels, with duplex stainless steels and other alloy steels [14]. The TIG method is also commonly used for joining stainless steels [15]. In general TIG welded joints can be characterized by the presence of undercutting and burn-through. The other defect that can appear during TIG joining is a lack of penetration. It was observed that weld depth can be increased by welding with the flux activated tungsten inert gas (ATIG) method [16,17].

In TIG welding, the torch introduces a high thermal noise into the measurement setup. Metal surfaces (including surfaces of nonferrous alloys and superalloys) have a small absorptivity and thus a weak emissivity in the wavelength range covered by typical uncooled IR cameras, totalling about 5% of the black-body emissivity. The radiance emitted by the metallic surfaces is weak. Consequently, the infrared images are blurry and faint. Additionally, there is the possibility of grease patches or oxidized zones and spatter that have different (higher) emissivity levels than the base material surface. In [18] this was the case for the water glass used to protect thermocouples from high temperature. These areas are often interpreted as hot spots that are considered damaged zones. Moreover, the high reflectivity of the metallic surface causes the phenomenon of parasitic reflection. In the case of welding, the heat from the hot torch, electrode, and welding arc is reflected by the surface, which acts as a mirror in the infrared spectrum. Reflected hot areas mask the real temperature on the material surface and complicate the interpretation of acquired thermograms. Some experts attempt to weaken any interference by using the shield plate or filter [19]. However, interference cannot be completely avoided. As the vast majority of new metallic materials, alloys and superalloys are used in harsh environments, are characterized by low thermal expansion coefficients, have relatively good weldability (cobalt, titanium, and nickel) and have low emissivity in the wavelength band used mainly by longwave infrared (LWIR) cameras (8–14 µm), so there is a constant demand for novel acquisition and processing methods that will limit these drawbacks.

Modelling of temperature distribution in welded joints by the finite element method (FEM) is widely applied. The main way to correlate and bound the simulation results with the experimental ones is the use of contact temperature measurements with the use of thermocouples [18]. It is a convenient method, but it has several drawbacks, such as a limited number of measurement points that can be set up on one workpiece and a small repeatability of thermocouple positioning. Additionally, use of thermocouples is not allowed in the production environment, because it requires previous preparation of samples and affects the quality of further product.

Large thermal gradients generated during welding due to localized heating by moving concentrated heat sources are causes of metallurgical changes, welding stresses, and distortion and can allow the formation of welding defects. 3D transient FEM numerical simulation is a proven and reliable method of analysis of thermo-metallurgical and mechanical effects of welding processes. 3D transient FEM welding simulations are computationally intensive, but offer a more detailed solution and greater application elasticity than analytical methods. Welding simulations do not converge 100% convergent with experimental results. However, the use of correct simulation methods, material models that factor in the change of thermal and mechanical parameters in the temperature range, and the correct simulation methodology significantly increase the accuracy of simulation [20,21,22,23].

In the paper a study on the possibility of clustering welded joint properties according to hardness and IR-based temperature measurements is presented. The key issue is the exact temperature measurement of the temperature that is proposed. According to well calibrated FEM simulation results, a valid ground truth temperature distribution was generated. Based on those distributions, a method of reflected temperature correction was proposed. Using corrected IR images, the condition of welded joints was clustered to differentiate the correct and incorrect joints from the hardness point of view.

## 2. Materials and Methods

The tests were carried out on thin sheets with a thickness of 1 mm of nickel superalloys type Inconel 625, welded by the TIG method. The sheets used to make the welds were from the Huntington Alloys Corporation industrial process (Huntington, WV, USA), which involved treating the material in a vacuum furnace. Next, a plastic processing was performed by cold rolling with intermediate heat treatment (recrystallization annealing). The chemical composition of the tested sheets is shown in Table 1.

**Figure 1:**
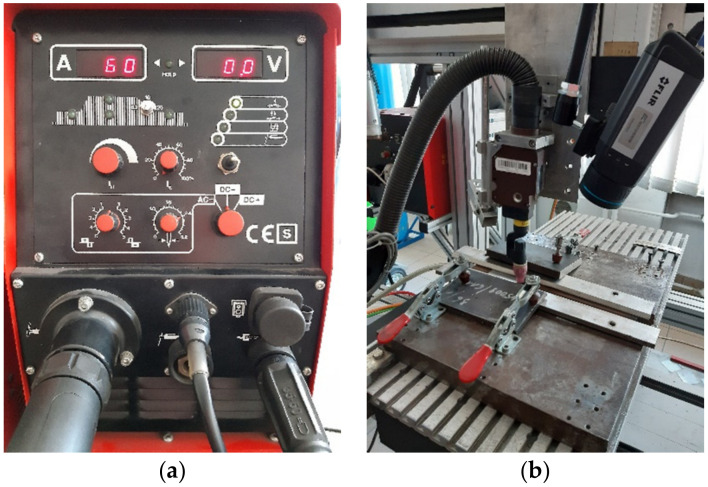
Welding device and TIG torch used during studies: (**a**) Casto-TIG 2002 device front panel, (**b**) TIG welding torch and FLIR A655sc IR camera mounted on the test stand.

The Casto TIG 2002 device (Castolin GmbH, Kriftel, Germany, Figure 1a) was used to weld sheets from the investigated Inconel nickel superalloy. The TIG welding of sheets was carried out under laboratory conditions with the following constant parameters: shield gas of Ar 12 L/min, ridge shield gas of Ar 3 L/min, tungsten electrode (thoriated) and WT20 with a diameter of 2.4 mm. Measurement of the temperature distribution on the surface of the welded joints was conducted with the FLIR A655sc infrared camera (IR CAM, spectral range of 7.5–14.0 µm) equipped with a 25 mm lens (Teledyne FLIR LLC, Wilsonville, OR, USA, Figure 1b). The spatial resolution of the camera was 640 × 480 px and the temporal resolution, as well as the acquisition frequency, was 60 Hz (60 fps). The optical axis of the sample camera was inclined to the plane of the sample at an angle of 30 degrees, and the distance between the camera and welded sample was 300 mm. For this length, the instantaneous field of view (IFOV) or size of one detector element in millimeters is 0.21. On the thermograms, the welding pool as well as the solidified and cooling joint area was acquired.

For the IR camera, a dedicated software, namely FLIR ResearchIRx64, (Teledyne FLIR LLC, Wilsonville, OR, USA) was used. The custom in house build PC used for the calculations was equipped with the following features: Intel Core i7-7700K, 4.2 GHz, ASUS TUF Z270 MARK 2 motherboard, and Corsair Vengeance LPX 16 GB DDR4 3000 MHz RAM. FEM simulations were performed in Visual-Weld 16.0 (SYSWELD core, ESI Group, Paris, France). Data post-processing and analysis was performed in the MATLAB R2021a environment with the use of an additional Teledyne FLIR Science File SDK (to read and process IR sequences).

Metallographic examination was performed in five stages: visual inspection of welded joints, macroscopic metallographic tests, observations of the structure on a light microscope, observations of the structure in a scanning electron microscope, hardness measurements. Visual tests of macroscopic welding imperfections of TIG-welded joints were carried out with the naked eye or at magnification up to 10×. The observed welding inconsistencies of the type: lack of penetration, burnout, undercut, etc. were classified according to the PN–EN ISO 6520-1 standard. Macroscopic metallographic tests were carried out on samples of joints cut perpendicularly to the weld line of the welded sheets. The cut samples were mechanically ground on abrasive papers with different granulations of the abrasive, successively from 100 to 800, and polished on cooled polishing wheels with water. To reveal the joint zone (JT) and the heat affected zone (HAZ), as well as the base material area (BM), the joint surfaces were etched with Adler’s reagent. with the following chemical composition: 3 g (NH_4_)_3_(CuCl_4_), 20 mL of distilled water, 50 mL of hydrochloric acid HCl, 15 g of iron chloride FeCl_3_. The etching of the samples was carried out in stages for 10 to 15 s at room temperature. Macroscopic observations of the etched joint samples were made using an OLYMPUS GX71 light microscope (Olympus Corporation, Tokyo, Japan) with a magnification of up to 50×. The width of the heat-affected zone and the dimensions of the face and ridge of the welded joints were determined by the metallographic method with a microscope magnification, which ensures the measurement with an accuracy of ±0.1 mm. Microscopic metallographic tests were performed on samples of welded joints perpendicular to the longitudinal axis of the weld. The samples were embedded in Duracryl Plus self-hardening resin (Metalogis S.C., Warszawa, Poland) and then mechanically ground on water-based abrasive papers with abrasive granulation of 320 to 1000. With each change of paper, the grinding direction was changed by 90°. The polished parts were polished on a Planopol-3 polishing machine (Struers A/S, Ballerup, Denmark) using a felt disc and a water suspension of Al_2_O_3_ (Struers A/S, Ballerup, Denmark). Polished samples of Inconel 625 superalloy joints were etched in a reagent containing, apart from HCl and HNO_3_ acid, approximately 2% HF acid. Etching was carried out several times at room temperature for 15 to 25 s. Metallographic observations were made on the OLYMPUS GX71 light microscope with a magnification in the range of 100 to 400×. All chemicals used for samples etching were manufactured by POCH, Avantor Performance Materials Poland S.A., Gliwice, Poland.

The hardness of the cross sections was measured using a Struers DuraScan 50 and Hauser hardness tester (Henri Hauser AG, Biel, Switzerland) with a force of 9.807 N, thus the hardness of 1 HV (or HV) was measured.

**Figure 2:**
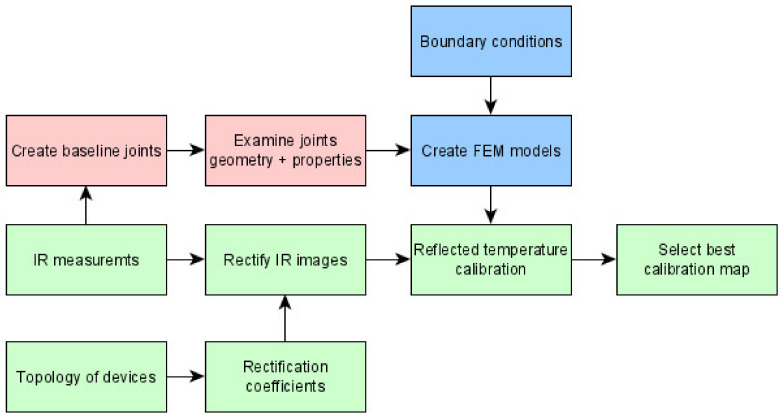
Diagram of reflected temperature correction in the IR image of TIG welding.

Correct assessment of the temperature for clustering is required. To overcome dynamic changes of apparent temperature caused by presence of reflected heat resulting in reflected temperature being a noise covering the correct temperature distribution. The heat source for this correct distribution is only the heated workpiece, and not the hot welding electrode, torch, and welding arc. The idea of a correction process is presented in Figure 2. The process requires information about the parameters of already made joints, thermogram sequences of those joints (taken during the process), and the topology of IR observing devices. Test joints are needed to make an FEM simulation that will reflect the results of the real process as best as possible.

FEM welding analysis is very susceptible to changes in simulation parameters input and, as such concept of heat source fitting—a process where parameters of moving heat source are determined based on some experimental data such as dimensions and shape of the fused zone, welding thermal cycle, or surface temperature distribution. TIG 3D welding simulations are most often carried out with a double ellipsoidal volumetric heat source (Goldak heat source) [24]. This heat source model accurately describes the temperature distribution in arc welding processes. The input parameters for the Goldak heat source are: power (given by welding energy per unit length multiplied by efficiency) and shape parameters width length and depth of the heat source [23,25].

**Figure 3:**
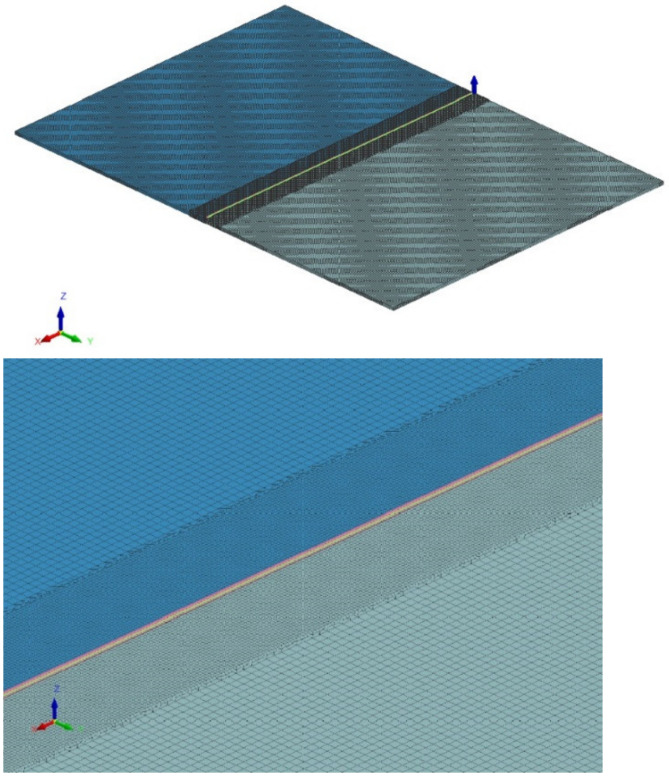
View of the meshed model and on the denser mesh in the area where the heat source model is applied.

The 3D FEM model was composed of 298,006 elements and 379,776 nodes. The 3D elements mesh had an increase in element density and a uniform hexahedron element size of 0.2 × 0.2 × 0.2 mm in the area where the heat source was applied. The value was chosen to fit the IFOV of IR image. The sides of the model were composed of 0.33 × 0.33 × 0.33 mm hexahedral elements and the transition layers were formed from pentahedral elements. The 3D element mesh and the area of mesh density are presented in Figure 3. The thermal boundary conditions were set to 20 °C free air of 20 °C exchange on all faces of the open element faces. The Goldak heat source was used. Energy per unit length was calculated from the registered TIG welding parameters. The heat source parameters resulting from the fitting of the heat source to the shape of the fused zone. Additional simulations with heat source thermal efficiency of 0.4 and 0.6 were performed. Other parameters of the heat source were the same as for each considered sample (welding parameters combination) regardless the thermal efficiency. The Inconel 625 material database used in simulation takes into account changes in thermal parameters such as density, thermal conductivity and specific heat in the range of 20–1300 °C. The commercial database provided with the SYSWELD software was used (database version/date 5 November 2014).

**Figure 4:**
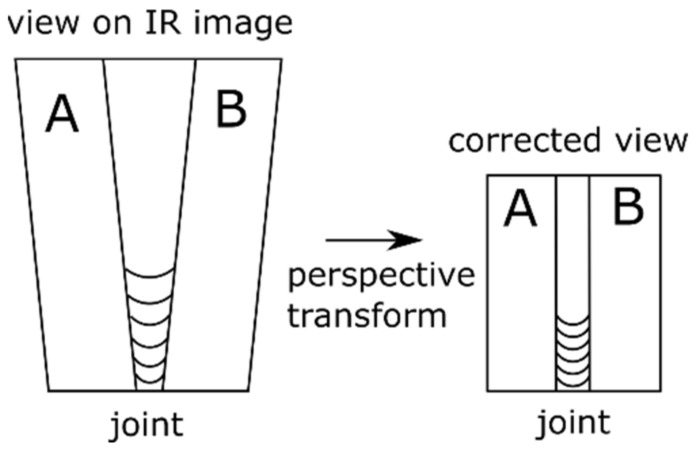
Idea of a projective (perspective) transform on the example of welding of two plates (marked as A and B). The piece on the original IR image is distorted, and after perspective transform the view is rectified (the viewing axis is perpendicular to the workpiece surface).

To apply results of FEM modelling to estimate the reflected temperature and, further, to calibrate IR images, it is necessary to remove the perspective that is introduced to images by the topology of the image acquisition setup. Perspective distortion (also called keystone distortion) is a common problem. It is especially apparent in an image with dominant vertical lines & shapes. The distortion is caused by the camera’s digital sensor (the focal plane) not being parallel to an object’s surface and/or not level with the centre of the object. If you shoot horizontally and level (perpendicular) with the centre of an object, its vertical lines will appear straight. If the camera is tilted up, it will bend inward towards the top of the picture. If the camera is tilted down, they will bend inward towards the bottom of the picture. The idea of this kind of transform is presented in Figure 4. It can be seen that after transformation in the resulting image, horizontal parallel lines are kept parallel. Vertical parallel lines are not parallel to each other after transformation. To achieve this result, a set of corresponding points must be found in both images. Then using a linear transform [26] the image is corrected. After this transform, a geometric alignment between the FEM based image of the temperature distribution and the IR image was set in order to produce a quality overlay of both images.

Thermograms after correction can be used to elaborate a reflected temperature correction map. For this operation, a constant emissivity ε = 0.13 was used. According to previous research, the emissivity of the Inconel 625 alloy is below ε = 0.2 [18]. After additional evaluations, it was found that it is even lower. The emissivity was checked at room temperature (20 °C) and at elevated temperature (100 °C). The test procedure consists of the painting a part of sample a black paint with known high emissivity (ε = 0.98). To perform measurements in the elevated temperature, the sample was heated in a controllable furnace. The temperature was then measured in a specially designed dark chamber, to avoid reflexes from external heat sources. Then the emissivity of the unpainted area was then changed iteratively to the moment, when the temperature of both material parts (painted and unpainted) was the same. The reflected temperature correction map was then calculated by minimizing the difference, understood as a mean squared error, between the FEM simulation results and the IR image. The range of possible reflected temperature TR was also narrowed down $T_{R}\in \langle 50;1500\rangle$ °C. Then, according to a greedy/exhaustive optimization algorithm, a parameter set consisting of the emissivity value and ten values of the reflected temperature were found. For the search procedure, the temperature step was set to $\Delta T_{R}=1$ °C. According to this procedure, a set reflected temperature-calibrated maps was defined. First maps were calculated for each test image, differing in the thermal efficiency of the reference FEM images. Additionally, an average map of all test samples was for consecutive thermal efficiencies (correct, that is, various for each sample, η = 0.4 and η = 0.6).

The corrected and rectified thermograms are then assessed. The distribution of temperature in a thermogram calculated over a line profile that is perpendicular to the weld axis is bounded with changes in hardness measured on a cross section of the joint taken at the same location as temperature. The third parameter is the welding liner energy calculated for a constant value of thermal efficiency, which is taken from previous experiments [18]. Linear energy of welding was calculated, using the following equation:

(1) $$E=\eta \frac{UI}{v}\;(J/\mathrm{mm})$$

where: η—thermal efficiency coefficient of the process (0.6 for the TIG process), *U*—welding current (A), *I*—arc voltage (V), *v* is the welding speed (mm/s).

All these values are then normalized to the range ⟨0;1⟩. It is needed to make distances between various quantities need to be comparable, because the range of temperature, hardness, and liner energy is different, thus a quantity with the largest span could significantly influence the clustering process. All this can lead to poor results of clustering. One of the open issues that needs to be solved is the number of clusters. The ability to distinguish correct joints, form incorrect ones, having e.g., low hardness in HAZ or joint is desired. Also, it is wanted to distinguish between joint and base material zones. The k-means clustering method was applied and the Manhattan distance metric was used [27]. Manhattan distance is preferred over the Euclidean distance metric as the dimension of the data increases; thus, for 3D data used in the clustering process of welded joints, it was applied. Both considered distances are used to measure the similarity of weld samples in the 3D space described by process parameters and joint properties. The Euclidean distance is a shortest, straight-line distance between two points in the 3D space (in considered case). It is calculated as the square root of the sum of the squared differences between two points. The Manhattan distance (called also city block distance) is the sum of the lengths of the projections of the line segment between the points onto the coordinate axes. It is calculated as the distance between real vectors using the sum of their absolute difference.

## 3. Results

**Figure 5:**
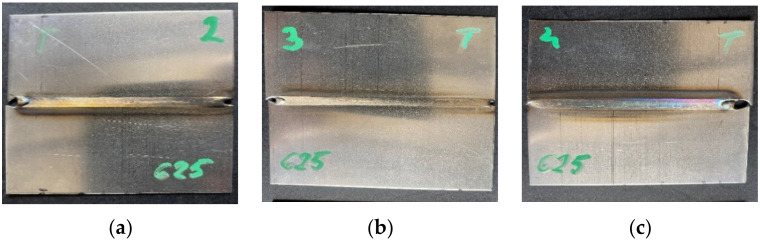
Weld faces made on Inconel 625 sheets, with current: (**a**) S2–40A, (**b**) S3–35A, (**c**) S4–45A.

There were two sets of test joints used in the investigation. The first was used to create a baseline for further clustering and to elaborate reflected temperature correction maps (Figure 5). The second was used for joint condition clustering.

### 3.1. Metalographic Examination

**Figure 6:**
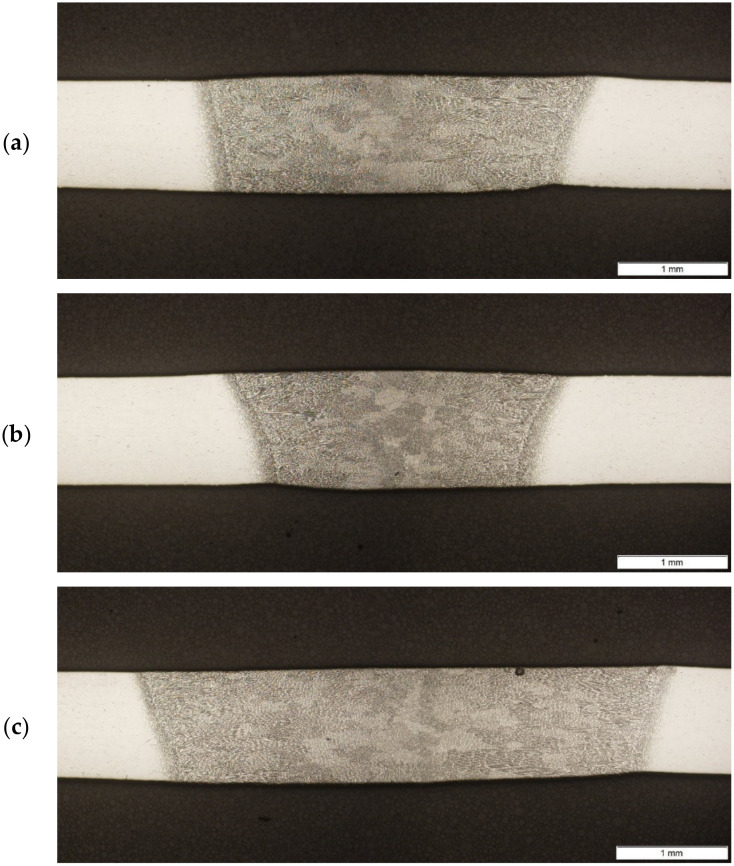
Macrostructure of cross-section of welded samples: (**a**) S2; (**b**) S3; (**c**) S4.

To model the heat source, which reflects real conditions and welding results, a registration process was carried out. Based on the geometry of the fused zone, simulation parameters were calibrated for three different samples made with different process parameters (Table 2). The heat-affected zone in the joints of Inconel 625 superalloys does not change significantly with the change in the linear energy of the arc. The estimated width of this zone is approximately 0.20 mm in the entire range of linear energy from approximately 68 J/mm to approximately 94 J/mm (Figure 6). On the other hand, the width of the weld face in this range of linear welding energy increases, respectively, from about 3.0 to about 4.9 mm, and the root from about 2.2 to about 4.1 mm (Figure 6). All energy values were calculated for constant thermal efficiency value η = 0.6.

For the purpose of hardness clustering, a second set of samples was used. These workpieces were also made from Inconel 625 alloy, but the thickness of the samples was 1.2 mm. Process parameters used to make those samples are gathered in Table 3. It can be seen, that those samples are characterized with higher linear energy, as it was the case for calibration sample (Table 2).

The minimum HAZ hardness of approximately 208 HV is demonstrated by welded joints with the highest linear arc energy of approximately 94 J/mm. With these welding parameters, a minimum hardness of the weld of approximately 240 HV was also achieved. In the intermediate zones of the tested joints, the hardness generally shows a differentiated dependence on the welding parameters that can be regarded as averaged values for the hardness of the adjacent zones.

**Figure 7:**
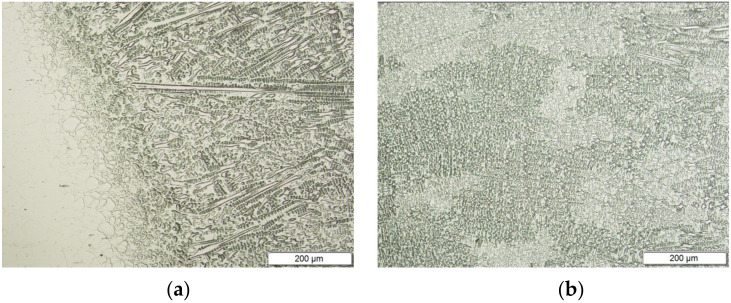
Structure of the joint made between Inconel 625 sheets (S3 sample): (**a**) HAZ—joint; zoom 200×; (**b**) joint, zoom 200×.

In the joint structure, the occurrence of narrow zones of columnar dendrites elongated in the direction of the fusion line and passing through is observed in them in large colonies of dendritic grains in the weld area of the weld (Figure 7a). On the other hand, in the heat-affected zones of these joints, the presence of fine grains of austenite was revealed. In the Inconel 625 superalloy sheets, a similar matrix structure is observed; however, the twin-austenite is finer-grained. The size of austenite grains determined by the microscopic method in Inconel 625 superalloy sheets is approximately 15 to 25 μm. In the heat-affected zone of the joints of Inconel 625 superalloys welded with a linear energy of approximately 68 J/mm, the austenite grain size of austenite is approx. 10 μm. On the other hand, welding these sheets with an energy of about 94 J/mm causes further, slight grain grinding in this zone. Fragmentation of the HAZ structure is undoubtedly related to the separation of intermetallic phases that block the movement of grain boundaries and thus inhibit their growth (Figure 7a).

**Figure 8:**
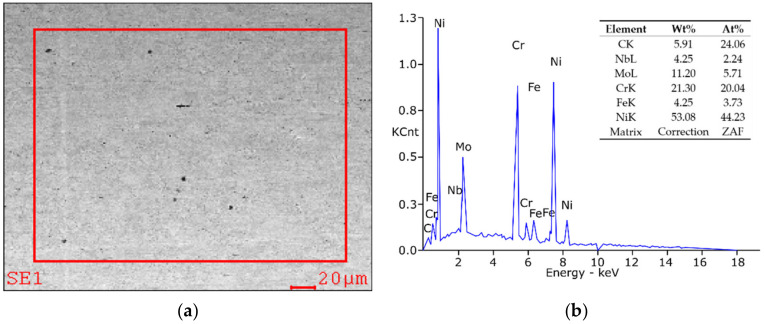
High-resolution transmission electron microscopy (HRTEM) image of the weld area in Inconel 625 sample: (**a**) view of the base material structure, (**b**) spectrogram of the quantitative analysis of the elements concentration, made by energy-dispersive X-ray spectroscopy (EDS).

The EDAX EDS microanalyzer (Ametec, EDAX, Mahwah, NJ, USA) was used for the microanalysis of the chemical composition of single precipitates revealed in the metallographic samples. X-ray microanalysis of the surface of base material and HAZ (Figure 8) showed in principle that the concentration of Cr, Fe and Nb is comparable to their participation in the chemical composition of the outstanding tested, while the concentration of carbon and molybdenum is increased to approximately 6% and approximately 11%, respectively, and the nickel concentration has fallen to approx. 53 ÷ 55%. A higher concentration of carbon in the tested welded zones disclosed by EDS may be bound, inter alia, with the presence of local carbide phases in the microanalysis area of microanalysis (Figure 8a), taken with SUPRA 35 (ZEISS AG, Oberkochen, Germany) by the reflected electron method at an acceleration voltage of 20 kV. The result of X-ray microanalysis confirms undoubtedly the presence in the HAZ of a combined carbide with M_2_C stoichiometry, namely (NbMo)_2_C.

**Figure 9:**
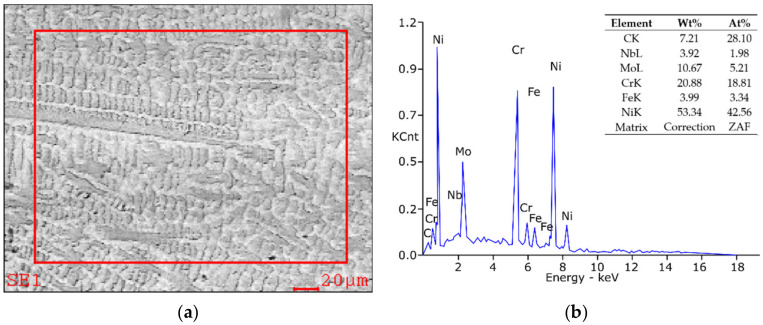
High resolution transmission electron microscopy (HRTEM) image of the weld area in Inconel 625 sample: (**a**) view of the dendritic structure in the joint, (**b**) spectrogram of the quantitative analysis of element concentration, made by energy-dispersive X-ray spectroscopy (EDS).

In the weld zone of the assumed welded joint, a primary, dendritic microstructure with clearly marked main axes of dendrites and precipitations in the form of eutectic, is revealed in interdendritic areas (Figure 9). The results of X-ray microanalysis from the joint surface of the joint showed basically identical distribution of the elements analysed, as in the case of the base material and HAZ of the tested joint (Figure 8b and Figure 9b). On the other hand, results of point X-Ray microanalysis of the structural components of the welds showed in the case of separation in the area of a dendrite, in the form of a local eutectic inflated concentration of carbon at 5.83%, niobium up to 18.40% and Mo up to 16.84% and Ti up to 0.77% at low Cr concentration of approx. 17%, Fe approx. 3% and Ni approx. 38%. Eutectic in this case can form an intermetallic phases of stable nitride (TiN) and more numerous MC composite carbides such as Nb(TiMo)C or M_6_C, such as, for example Mo(Ni)_6_C with an isomorphic structure (FCC) of isomorphic to the solution γ, but with a lower crystallization temperature.

### 3.2. FEM Modelling

**Figure 10:**
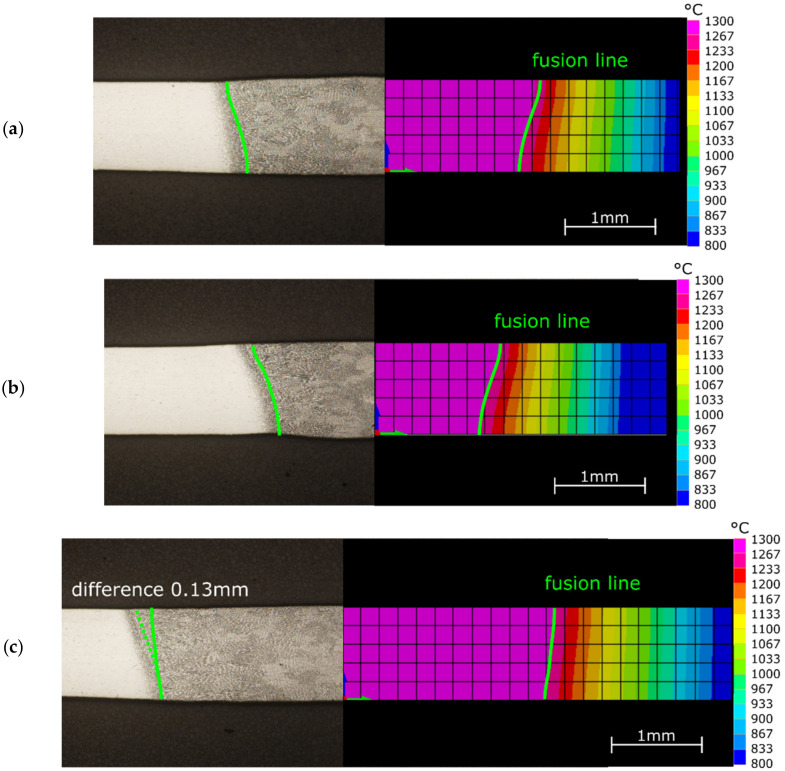
Macrostructure of the cross section of welded samples: (**a**) S2; (**b**) S3; (**c**) S4.

According to the procedure described in Section 2 a set of simulations was elaborated. First, for samples S2–S4, the simulation (properties of heat source) was calibrated in a way that reflects real welding conditions in the model. The simulation parameters were changed iteratively to obtain the geometry of the temperature field cross section, which will have the best correspondence to the weld and HAZ areas. The fusion zone (FZ) was represented as a diagram of the maximum temperature value on the cross section, located midway along the length of the joint. In this case, the white area representing the temperature that exceeds the weld melting point marks the boundary of the weld (Figure 10). Both the shape and size of FZ and HAZ computed for samples T2 and T3 are in good agreement with the measurements. The T4 sample differs from the original real sample mainly in the shape of FZ on the weld root side. The width of HAZ is also correctly reflected in the FEM results, because for all considered samples it was measured on a level of 0.2 mm. Taking into consideration that the temperature distribution only on the weld face side will be considered, the obtained correspondence can be regarded as sufficient.

The heat source parameters for consecutive simulations are gathered in Table 4. It can be seen that for different samples the thermal efficiency varies from η = 0.43 to η = 0.505. It is a relatively large span. Furthermore, it differs from the commonly used value for TIG ($\hat{\eta }$ = 0.6). For each sample, results were taken, when the heat source was in 25, 50 and 75% of workpiece length.

**Figure 11:**
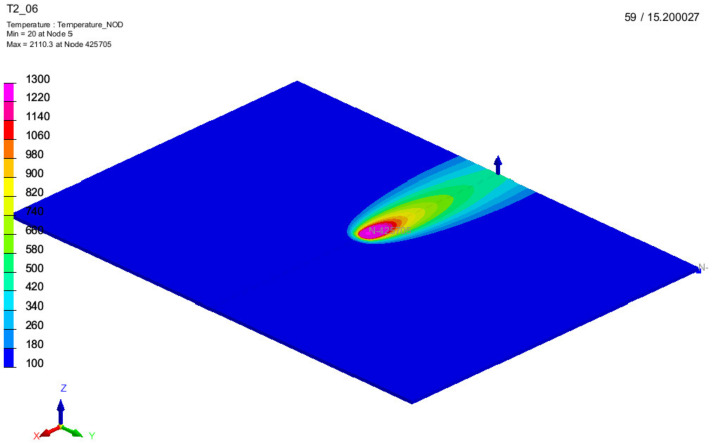
Exemplary result from FEM simulation results in SYSWELD software; S2 with thermal efficiency $\hat{\eta \;}\;$= 0.6.

**Figure 12:**
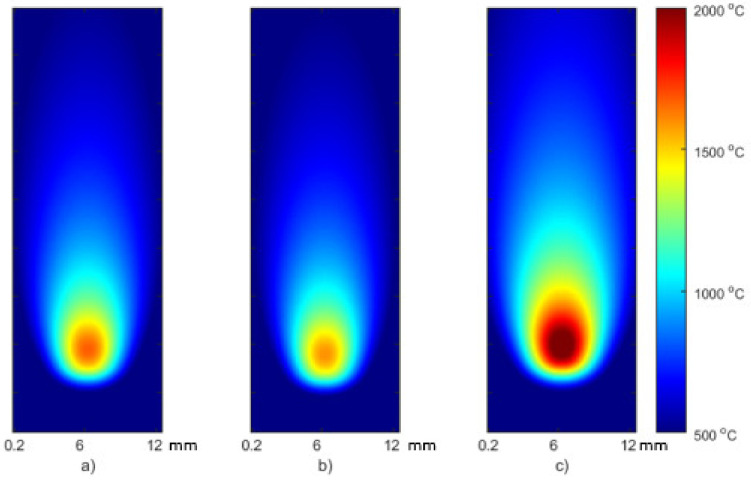
FEM simulation results for S2: (**a**) calibrated thermal efficiency η = 0.43, (**b**) η = 0.4, (**c**) η = 0.6. Results from SYSWELD visualized in MATLAB.

The simulation results were the temperature distribution in the 3D model in specific time points (time cards), Figure 11. The length of each time card was automatically adjusted automatically based on thermal gradients, and the results were in the range 121–187 time cards long. All simulation results were exported from SYSWELD and later processed in the MATLAB environment. In Figure 12 a comparison of simulation results generated for the S2 sample parameters. The temperature pattern depends on the thermal efficiency. It can be seen that for higher efficiency, more heat from source is transferred to the material and a higher temperature can be observed on the material surface. The difference between maximal temperature for the most extreme values of thermal efficacy exceeds 500 °C (Figure 11).

### 3.3. Reflected Temperature Maps Generation and IR Image Correction

**Figure 13:**
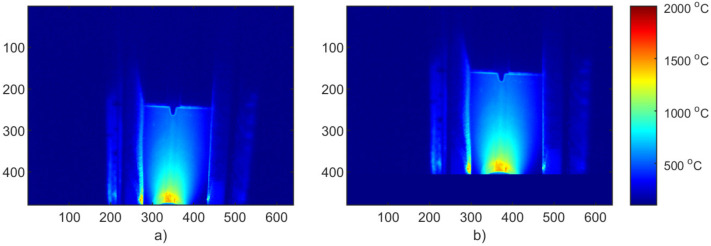
Exemplary IR image from the S2 sequence: (**a**) raw, (**b**) rectified.

To generate reflected temperature correction distribution map, first it was necessary, to unwarp the IR image to reduce the influence of perspective on geometry of objects visible on each image. The necessary coefficients were calculated according to the geometry of the vision system geometry. The results of image rectification are presented in Figure 13. It can be seen that after rectification the face surface of welded workpiece is ideally projected on the image pane (Figure 13b). Moreover, the right metal part that was used to hold and stabilize the welded elements is ideally parallel to the joint axis.

**Figure 14:**
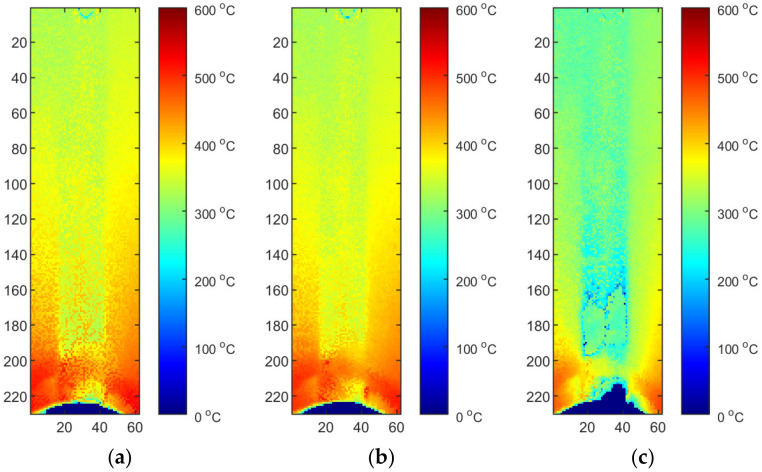
Calibration maps for S2–M_2_: (**a**) calibrated thermal efficiency (η = correct), (**b**) η = 0.4, (**c**) η = 0.6.

**Figure 15:**
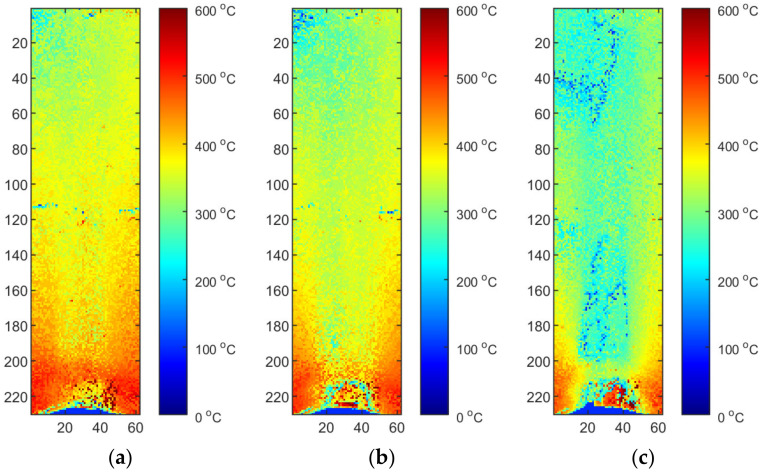
Averaged calibration maps for S2/S3/S4 sequences—M_AVG_: (**a**) calibrated thermal efficiencies (different for each IR test IR image sequence, η = correct), (**b**) η = 0.4, (**c**) η = 0.6.

**Figure 16:**
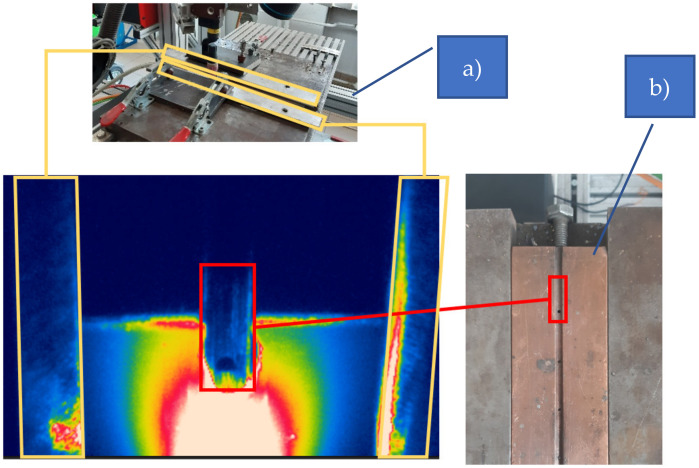
Location of artifacts caused by (**a**) holders and (**b**) gas nozzles.

Figure 14 and Figure 15 present correction maps for thermograms coming from one sequence (S2) and averaged maps. In both, there is a general pattern visible. Because of the way the welded workpiece was mounted, namely, it was sealed on both sides, with metal plates, a relatively large reflection is on both sides of a joint. In the middle there was an insert with drilled holes through which gas was supplied to protect the root of the weld. This area is cooler than areas on both sides. As was supposed, most of the reflections are concentrated in the immediate distance from the welding torch outlet. Averaging of correction maps tends to produce a more complex solution that can be treated as a more general solution. It is visible, that it generalizes sequence-dependent artifacts, like disturbances by tor outlet or specific shape of gas nozzles in the middle of the welding pad (Figure 16).

Results of the FEM simulation were generated for three time points in which the electrode tip reached the position at 25%, 50% and 75% of the workpiece length. Knowing the moment of welding start in the IR sequence and the welding speed, thermograms corresponding to specific timepoints were found. For all sequences (Table 2), three test images were selected and later subjected to reflected temperature correction.

**Figure 17:**
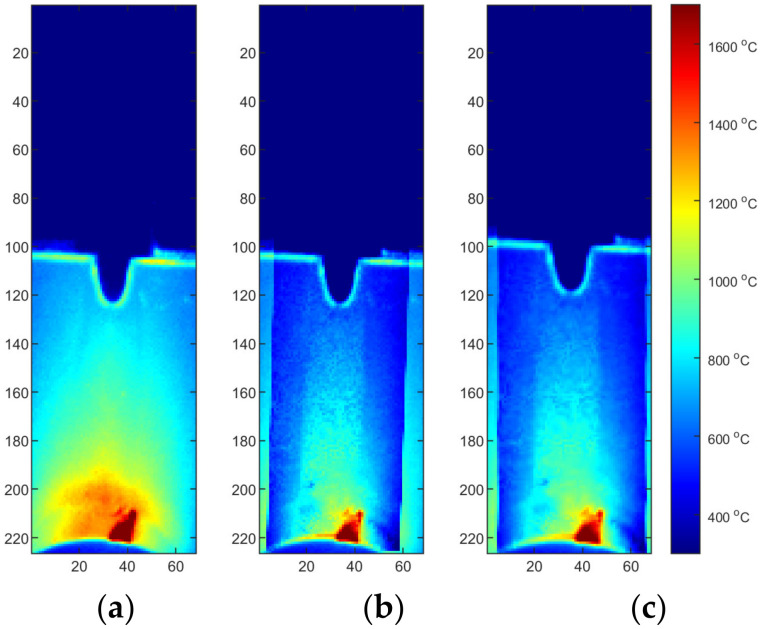
Thermograms of the S2 thermogram id = 376 (50% length): (**a**) original IR image; (**b**) calibrated image, η = correct, M2, (**c**) calibrated image, η = correct, M2, and rectified.

**Figure 18:**
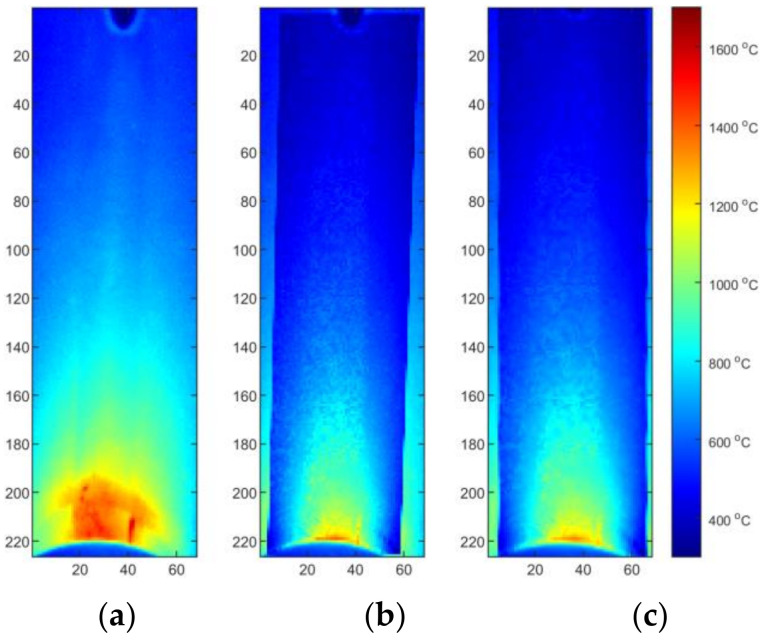
Thermograms of the S2 thermogram id = 603 (75% length): (**a**) original IR image; (**b**) calibrated image, η = correct, M2, (**c**) calibrated image, η = correct, M2, and rectified.

**Figure 19:**
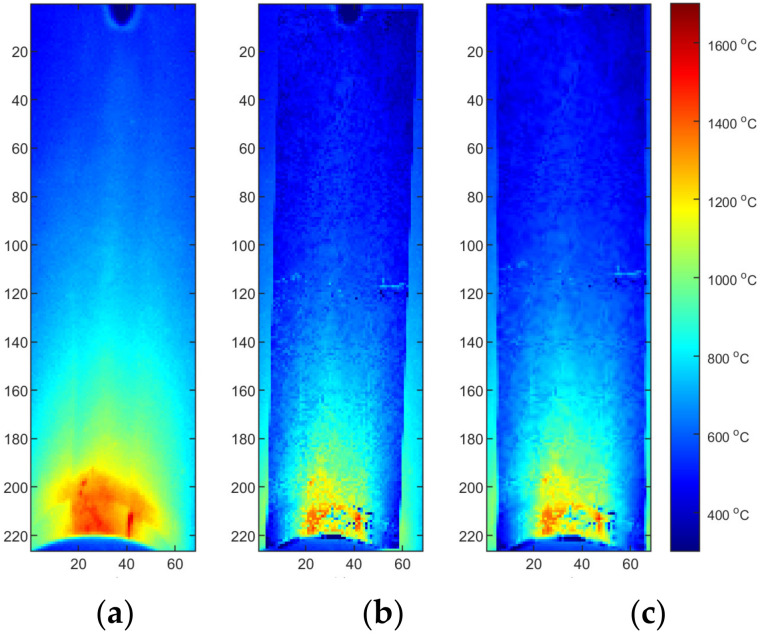
Thermograms of the S2 thermogram id = 603 (75% length): (**a**) original IR image; (**b**) calibrated image, η = correct, MAVG, (**c**) calibrated image, η = correct, MAVG, and rectified.

Two reflected temperature correction strategies were evaluated. In the first one for each sequence that reflects a certain set of process parameters (S2/S3/S4), a dedicated correction map was used. In this case, each image tends to be as close to the FEM image as possible. From the qualitative point of view, this approach is susceptible to decreasing of artifact distinctness. It can be seen that small hot spots that are visible in the solidified weld area (Figure 17a and Figure 18a) are barely visible in corrected thermogram (Figure 17b,c and Figure 18b,c). On the other hand, as the span between hottest and the coolest points in a thermogram decreases after correction, the change of the representation range, and the redrawing of IR images in the new scale reveal most of artifacts that justify presence of weld defects and inconsistencies. The second strategy consist of applying an averaging over all calibration maps and using this averaged reflected temperature distribution map (Figure 19). It is noticeable that the shape of area with increased temperature is not as smooth as it was in the case of map dedicated for one sample. The small asymmetry of temperature near the torch is preserved and the temperature is in a similar range as it is in Figure 18. For both types of correction maps extracted for ground truth FEM simulations generated for heat effectiveness, 0.4, 0.6 and the correct value were used.

To quantify the results of thermogram correction, a mean absolute error (MAE) was used. It measures the difference between thermogram (original/raw or corrected) and FEM simulation result:

(2) $$\mathrm{MAE}=\frac{1}{n}\;\sum_{i=1}^{n}\left|T_{OBS,i}-T_{FEM,i}\right|$$

where: $T_{OBS,i}$ is the temperature in pixel *i* in thermogram and $T_{OBS,i}$ is the temperature in pixel *i* in temperature map, being the result of FEM simulation.

All results were collected in Table 5. It can be seen that it was possible to obtain an error level less than 10 °C. Having the map calculated for valid thermal efficacies (η = correct, M_AVG_) it was possible to obtain a best average MAE for all thermograms tested. The average $\hat{\mathrm{MAE}}$ = 8.9 °C, what is a more than a satisfactory result. It has to be emphasized that all results regardless of the correction map using which they were generated were compared to FEM simulations performed with experimentally tuned parameters.

**Figure 20:**
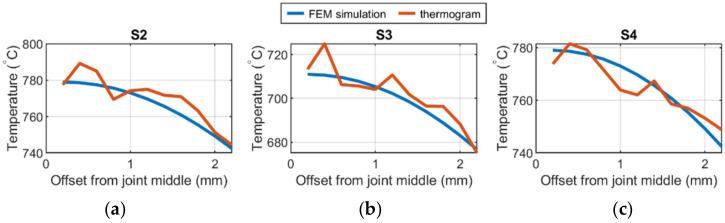
Comparison of the temperature profiles of IR image and FEM simulation for η = correct MAVG mapping for the 75% of the length for samples: (**a**) S2; (**b**) S3; (**c**) S4.

The application of the correction map that gives best results is visualized in Figure 20. There are two plots in each axis: FEM temperature (blue) and corrected IR temperature (red). It can be easily assessed that applying map (η = correct, M_AVG_) leads to a temperature distribution that is close to the ideal distribution. There are two main reasons for the occurrence of deviations:

- presence of point or area temperature disturbances that are the results of welding process instabilities or weld faults,
- uneven distribution on the reflected temperature in certain regions of the correction map caused by temperature artifacts occurring on the surface of samples that were used for correction maps generation.

**Figure 21:**
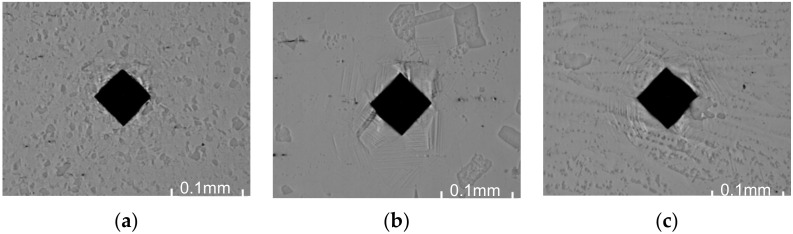
Idents for Vickers hardness measurements made in different areas of sample S17, magnification 10×: (**a**) base material; (**b**) HAZ; (**c**) joint.

Finding that the averaged correction map is less selective in preserving small, point changes in temperature but still having the low overall MAE, it was decided to apply it on a second set of IR images, gathered for a different set samples. These samples were also made from Inconel 625 but all sheets were 1.2 mm thick. For that, a different set of welding parameters was used to create joints (Table 3). For all samples, hardness was measured in row, and consecutive idents were separated by 0.4 mm. Exemplary identifications for base material, HAZ, and joint are presented in Figure 21.

To compare hardness results with the temperature, a part of measurements covering the limited number of samples counting form the weld axis (Figure 20) is covered. It can be seen that for the S14 and S20 hardness is almost the same in all zones. Thus, for combinations of parameters: 60 A, 3 mm/s and 70 A, 7 mm/s, the amount of heat transferred into the welding pool is similar. For 70 A welding speed, decreasing leads to widening of joint zone and slight decrease of hardness in joint and HAZ.

**Figure 22:**
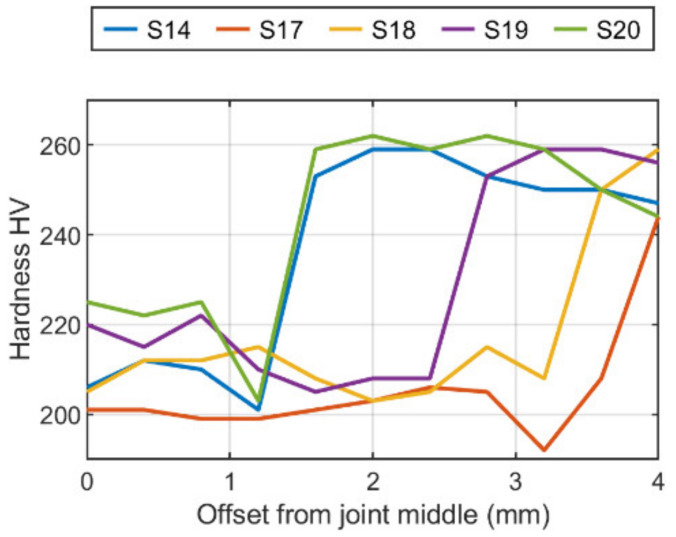
Hardness distribution for samples S14, S17–S20 for 25% of length as a function of offset from the joint axis.

**Figure 23:**
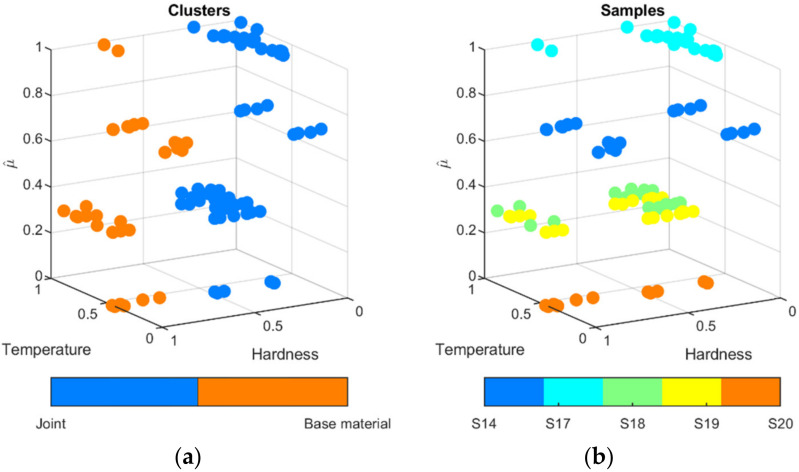
Clustering results for two groups on temperature, hardness and linear energy estimate $\hat{µ}$ (**a**) and class distribution (**b**).

**Figure 24:**
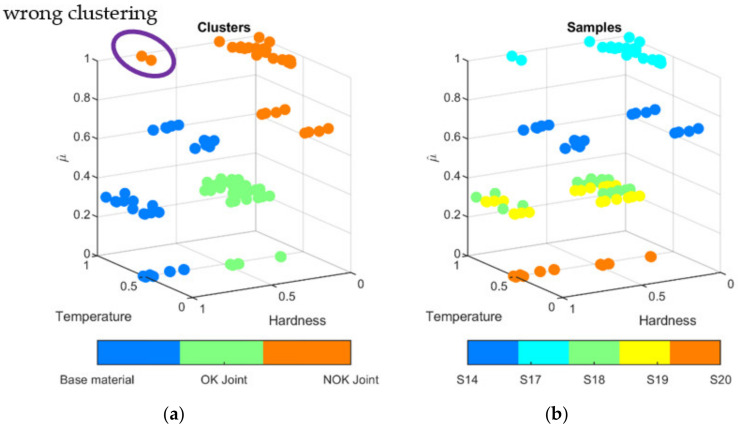
Clustering results for three groups on temperature, hardness and linear energy estimate $\hat{µ}$ (**a**) and class distribution (**b**).

The clustering process was applied to distinguish automatically between the correct and incorrect processes that is resulting with excessive lowering of the hardness in joint. To group the measurement values of hardness, temperature were bounded. To do this, consecutive hardness measurements (Figure 22) were connected with corresponding temperature values measured on the surface of welded sample (Figure 19). For each sample, 22 value pairs for two positions were obtained. Moreover, the third parameter, the liner energy was applied. As the voltage remains almost constant, it was set to 11 V. The thermal efficiency was set to η = 0.4 according to previous results (rounded down thermal efficiency obtained for considered test samples S2–S4, Table 4). All triplets $\left\{t,\;\mathrm{HV},\;E\right\}$ were projected into 3D space (Figure 23b and Figure 24b). It can be seen that differences between individual test samples are visible. In general, higher linear energy results in higher temperature and lower hardness in joint. Applying the clustering for two clusters leads to the creation of two groups (Figure 23a). Those groups reflect only base material and joint. Adding the third cluster, an additional separate group can be formed. After that, joints can be differed in terms of properties corrections is joints (Figure 23b). Groups for correct (OK joint) and incorrect (NOK joint) are almost perfectly gathering valid samples. Only two points of the base material (marked purple oval, Figure 23a) are incorrectly clustered. Adding the fourth cluster is not a solution to overcome this drawback. For this clustering procedure, adding new sample will lead to assignment to proper cluster. Thus, an easy way of distinguishing between correct and incorrect joints is possible.

## 4. Summary and Conclusions

In the paper a method of correction that is based on the calculation of the difference between results of the FEM simulation and IR images is described and validated. With highly accurate temperature distributions on the workpiece surface it was possible to automatically cluster weld conditions in the joint areas. When clustering to three groups a differentiation between base material and proper and improper joint was possible.

In [18] a regressive model was elaborated and used to predict the hardness values in joints. The mean prediction error for the hardness of joint area at the level of err = 1.25%. Nevertheless, based only on the relative temperature changes, it is difficult to obtain an exact correspondence between process parameters and joint properties. Moreover, it was proven that relying only on a constant and collected form literature review value of thermal efficiency to calculate linear energy of welding can be misleading. It was found during FEM modelling that for wrong setup of heat source parameters, like thermal effectiveness, errors in the temperature field distribution can exceed several hundred °C. Generally, from the practical point of view, it can be stated the key issue is the elaboration of high quality FEM model, that address all properties of correct weld made to join elements made of certain material using chosen technology. In the paper a limited approach was used, that was based only on bounding the simulation results with the shape of real welded joint and HAZ cross-section. As FEM modelling is generally used to predict the stress distribution in welded joints [28,29], it was applied in the research to obtain a reliable and accurate temperature distribution on the surface of the workpiece. Even using this limited approach it was found, that naïve application of theoretic values of thermal efficiency can lead unexpected results, when the temperature distribution remains unreliable. Nevertheless correction of the reflected temperature based on FEM simulation was found to be more accurate than using thermocouples [18,30]. Using the reflected temperature calibration method based on optimal FEM simulation that was proposed in this paper allows temperature measurements in solidified welded joint area with an average MAE lower than 10 °C. Such a small measurement error makes it possible to perform further investigations on additional properties of joint clustering and predictions, such as impact strength or residual stress. For performed studies clustering error for faulty weld was on a level of about 7%. There were no faulty samples clustered as correct ones, but further investigation is needed to confirm the generalization ability of the proposed approach.

The proposed approach has also several drawbacks limiting the applicability. First it is strongly dependant on the geometry of the measurement system. To ensure a proper correspondence between the temperature distribution in FEM nodes and IR images a perspective transform is required. To get correct results the parameters of this transform must be tuned carefully, which is difficult because there is no generally no reference image that can be used to evaluate the quality of the projective transform. Only the desired dimensions of welded workpiece can be calculated analytically and used as a ground truth for any performed transformation. On the other hand, the quality and validity of summation results should be proven using temperature measurements. As it was already mentioned the use of thermocouples is problematic. Other possible solution is use of two-colour pyrometers, that are measuring temperature for unknown emissivity of materials surface. This demands a special device to perform the measurements, that can often only measure temperature in one spot at the time, what can be insufficient to confirm the correctness of FEM-based temperature distributions. In addition, a FEM simulation has to be made for each combination of material type, workpiece dimension and process parameters. This operation must always be preceded by the fabrication of reference welds.

## Figures and Tables

**Table 1:** Chemical composition of the investigated Inconel 625 superalloys.

Super- Alloy	Element Concentration, wt %													
	Ni	Cr	Fe	Mo	Nb	Co	Mn	Cu	Al	Ti	Si	C	S	P
Inconel 625 *	60.7	21.76	4.27	8.96	3.56	0.07	0.07	-	0.14	0.18	0.08	0.01	0.0003	0.007

* Nb+Ta—3.56%, N—0.01%.

**Table 2:** Parameters of the welding processes selected to make sample joints.

Sample ID	Current (A)	Welding Speed (mm/s)	Linear Energy (J/mm)
S2	40	3	81.6
S3	35	3	68.58
S4	45	3	94.5

**Table 3:** Parameters of the welding processes selected to make sample joints.

Sample ID	Current (A)	Welding Speed (mm/s)	Estimated Energy per Unit Length (J/mm)
S14	60	3	132
S17	70	3	256.6
S18	70	5	154.7
S19	70	5	154.7
S20	70	7	110

**Table 4:** The heat source parameters resulting from the fitting of the heat source fitting to the shape of the fused zone.

Simulated Variant	Energy per Unit Length (J/mm)	Thermal Efficiency (η)	Width (mm)	Length (mm)	Depth (mm)
S2	136.0	0.43	3.5	3.5	0.25
S3	114.3	0.44	3.0	3.0	0.25
S4	157.5	0.505	4.5	4.5	0.25

**Table 5:** Mean absolute error (MAE) of the temperature distribution in IR images sets compared to ideal distribution in the FEM simulation results.

	η = Correct				η = 0.4				η = 0.6			
	M_AVG_	M_S2_	M_S3_	M_S4_	M_AVG_	M_S2_	M_S3_	M_S4_	M_AVG_	M_S2_	M_S3_	M_S4_
S2	9.9	11.7	**7.4**	12.9	11.0	18.1	12.9	33.0	31.9	46.6	23.9	9.9
S3	11.0	23.8	29.5	16.5	70.9	58.7	73.2	115.9	7.4	5.2	**2.2**	9.8
S4	**5.7**	17.0	15.9	7.2	14.9	23.9	10.1	19.6	13.1	12.3	16.9	44.7
avg.	**8.9**	17.5	17.6	12.2	32.3	33.6	32.1	56.2	17.5	21.4	14.3	21.5

## Data Availability

The data presented in this study are available on request from the corresponding author. The data are not publicly available because the authors do not wish to publish additional material.
